# Implementation of a Robotic Surgical Program in Gynaecological Oncology and Comparison with Prior Laparoscopic Series

**DOI:** 10.1155/2015/814315

**Published:** 2015-02-15

**Authors:** Natalia Povolotskaya, Robert Woolas, Dirk Brinkmann

**Affiliations:** Portsmouth Cancer Centre, Queen Alexandra Hospital, Portsmouth, UK

## Abstract

*Background*. Robotic surgery in gynaecological oncology is a rapidly developing field as it offers several technical advantages over conventional laparoscopy. An audit was performed on the outcome of robotic surgery during our learning curve and compared with recent well-established laparoscopic procedure data. *Method*. Following acquisition of the da Vinci Surgical System (Intuitive Surgical, Inc., Sunnyvale, California, USA), we prospectively analysed all cases performed over the first six months by one experienced gynaecologist who had been appropriately trained and mentored. Data on age, BMI, pathology, surgery type, blood loss, morbidity, return to theatre, hospital stay, and readmission rate were collected and compared with a consecutive series over the preceding 6 months performed laparoscopically by the same team. *Results*. A comparison of two consecutive series was made. The mean age was somewhat different, 55 years in the robotic versus 69 years in the laparoscopic group, but obesity was a feature of both groups with a mean of BMI 29.3 versus 28.06, respectively. This difference was not statistically significant (*P* = 0.54). Three subgroups of minimal access surgical procedures were performed: total hysterectomy and bilateral salpingooophorectomy (TH + BSO), total hysterectomy and bilateral salpingooophorectomy plus bilateral pelvic lymphadenectomy (TH + BSO + BPLND), and radical hysterectomy plus bilateral pelvic lymphadenectomy (RH + BPLND). The mean time taken to perform surgery for TH + BSO was longer in the robotic group, 151.2 min compared to 126.3 min in the laparoscopic group. TH + BSO + BPLND surgical time was similar to 178.3 min in robotic group and 176.5 min in laparoscopic group. RH + BPLND surgical time was similar, 263.6 min (robotic arm) and 264.0 min (laparoscopic arm). However, the numbers in this initial analysis were small especially in the last two subgroups and do not allow for statistical analysis. The rate of complications necessitating intervention (Clavien-Dindo classification grade 2/3) was higher in the robotic arm (22.7%) compared to the laparoscopic approach (4.5%). The readmission rate was higher in the robotic group (18.2%) compared to the laparoscopic group (4.5%). The return to theatre in the robotic group was 18.2% and 4.5% in laparoscopic group. Uncomplicated robotic surgery hospital stay appeared to be shorter, 1.3 days compared to the uncomplicated laparoscopic group, 2.5 days. There was no conversion to the open procedure in either arm. Estimated blood loss in all cases was less than 100 mL in both groups. *Conclusion*. Robotic surgery is comparable to laparoscopic surgery in blood loss; however, the hospital stay in uncomplicated cases appears to be longer in the laparoscopic arm. Surgical robotic time is equivalent to laparoscopic in complex cases but may be longer in cases not requiring lymph node dissection. The robotic surgery team learning curve may be associated with higher rate of morbidity. Further research on the benefits to the surgeon is needed to clarify the whole picture of this versatile novel surgical approach.

## 1. Background 

Since the first laparoscopic hysterectomy minimal access surgery (MAS) has become a standard of care in gynaecological oncology as it is associated with quicker recovery [1]. In addition, the technology and range of minimal access surgery skills have expanded to the level of radical hysterectomy and pelvic and para aortic lymphadenectomy as a routine practice in many centres [2, 3].

In gynaecological oncology the incidence of endometrial cancer which is primarily treated with surgery, is increasing through the obesity epidemic, which also contributes to multiple comorbidities in these patients which in turn places significant limitations on the feasibility of performing surgery by the laparoscopic approach.

In April 2005, Reynolds et al. reported on a preliminary series of  7 robotic total hysterectomies with bilateral salpingooophorectomy and pelvic lymphadenopathy for endometrial cancer [4]. A further 30 cases of robot-assisted hysterectomy for endometrial cancer were reported by Marchal et al. [5]. A live telecast of a robotic hysterectomy with staging for endometrial cancer was performed in 2007 and introduced robotic surgery to a wider audience [6]. Consequently, interest in robotics for the management of gynaecologic cancers expanded.

Robotic surgery in gynaecological oncology is a rapidly developing field as it offers technical advantages over conventional laparoscopy [7]. The results of many studies indicating a shorter stay, decreased blood loss, lower transfusion rate, and lower conversion to laparotomy rate, and adequacy of surgical staging [8–13] are in favour of the robot over the laparoscopic route. In addition, improved surgical field visualization, superior ergonomics, instrument articulation, decreased tremor, and apparently shortened learning curve make robotic-assisted surgery potentially advantageous [9–14]. In the morbidly obese patients who present a significant challenge for laparoscopic and open surgery, robotic surgery has the potential for decreased postoperative complications [15]. Also, it is possibly beneficial for the elderly patients with endometrial cancer who may not be able to tolerate a steep Trendelenburg position and the high pressure needed for abdominal insufflation due to their comorbidities. These demands are less in robotic surgery [14, 16–18], making MAS feasible for this group of patients who otherwise may have had open surgery. This is encouraging as so far no obvious difference in survival has been reported in the patients who have undergone robotic and laparoscopic surgery for endometrial cancer [19].

The benefit of the robotic surgery to the surgeon is not widely considered. Although laparoscopic procedures significantly benefit patients in terms of decreased recovery times and improved outcomes, they contribute to mental fatigue and musculoskeletal problems among surgeons [20–22]. Musculoskeletal injury to the surgeons can lead to a significant National Health System financial loss. The published evidence is that 87-88% of surgeons who regularly perform minimally invasive surgery suffer from occupational symptoms or injuries which are primarily high case load-associated [23, 24]. The financial losses due to time off related to the injury are significant especially taking into consideration the length and expense of surgical training [25, 26]. Such prevalent occupational strain presents a growing problem in the face of increasing demand for MAS. Robotic surgery with its better ergonomics may reduce this problem [24].

The higher cost [27–29] remains a limiting factor in the development of robotic surgery worldwide. This technique once established may be helpful in reducing the occupational hazards to the minimal access surgeons, improving their efficiency and decreasing financial losses due to time off and hence increasing the case load. Surgeon preferences for robotics may allow some women to undergo a minimally invasive procedure who may otherwise have undergone laparotomy.

The aim of our study was to determine whether robotic surgery during the learning curve of the team has comparable parameters of time and morbidity when compared to the well-established laparoscopic approach.

## 2. Methods

Following acquisition of da Vinci Surgical System (Intuitive Surgical, Inc., Sunnyvale, California, USA), we prospectively analysed all gynaecological robotic cases performed over the first six months (22 cases). All the cases were performed by one experienced gynaecological oncologist who had been appropriately trained and mentored in robotic surgery who was supported by a trained robotic surgery team.

Data on age, body mass index (BMI), pathology, surgery type and timing, blood loss, morbidity, return to theatre, hospital stay, and readmission rate were collected and compared with a consecutive series over the preceding 6 months performed laparoscopically by the same team (22 cases). These data were prospectively collected via our hospital electronic database.

The operative time was defined as the time from the incision to skin closure. Hospital stay was calculated in the hours from the time of admission to the discharge and converted subsequently into days. Complications were divided into intraoperative and postoperative. Any conversion to an open procedure, return to theatre, and readmission within 30 days of surgery were noted. Intraoperative complications were defined as bowel, bladder, ureteric, nerves injuries (including those related to the positioning of the patient on the operating table), and vascular injury. Postoperative major complications were defined as those necessitating intervention by Clavien-Dindo (grades 2 and 3) classification. Mild complications (Clavien-Dindo grade 1), such as, for example, slight wound infection or mild urinary tract infection, were not included in the analysis as MAS techniques were associated with early discharge from hospital leading to the situation that they may not be captured by the hospital records.

## 3. Results

A comparison of two consecutive series is presented in Table 1. The mean age was somewhat different, but obesity was a feature of both groups with a mean BMI 29.3 (robotic group) versus 28.06 (laparoscopic group). This difference was not statistically significant (*P* = 0.54). Three subgroups of minimal access surgical procedures were performed: total hysterectomy and bilateral salpingooophorectomy (TH + BSO), total hysterectomy and bilateral salpingooophorectomy plus bilateral pelvic lymphadenectomy (TH + BSO + BPLND), and radical hysterectomy plus bilateral pelvic lymphadenectomy (RH + BPLND). The mean time taken to perform surgery for TH + BSO was longer in the robotic group, 151.2 min compared to 126.3 min in the laparoscopic group. TH + BSO + BPLND surgical time was similar to 178.3 min in robotic group and 176.5 min in laparoscopic group. RH + BPLND surgical time was similar, 263.6 min (robotic arm) and 264.0 min (laparoscopic arm). However, the numbers in this initial analysis were small especially in the last two subgroups and do not allow for statistical analysis.

There was no conversion to the open procedure in either arm. Estimated blood loss in all cases was less than 100 mL in both groups.

The rate of complications was higher in the robotic arm (22.7%) (Table 2) compared to the laparoscopic approach (4.5%). In the laparoscopic arm, there was one case of infected haematoma in a 90-year-old, obese patient who underwent TH + BSO for endometrial cancer, who was readmitted and underwent laparoscopic wash out. She made a good recovery.

In the robotic group, there were three infected pelvic haematomas, who were readmitted a few days later, one of which (RH + BPLND case) underwent laparoscopic wash out performed by the on-call team which was counted as a return to theatre and the other 2 were managed with antibiotics.

Also an anterior compartment syndrome of the lower leg occurred, which developed in the immediate postoperative period in a patient known to have sickle cell trait (RH + BPLND case). The patient was immediately operated on by a plastic surgical team. She subsequently required skin grafting but recovered well.

Furthermore, a known immunocompromised patient who had RH + BPLND presented with a retroperitoneal abscess on day 15 after surgery. She had a protracted course originally managed with antibiotics followed by bilateral radiological drainage after which bowel injury was noted in the caecum and descending colon. That led to return to theatre for a laparoscopic ileostomy. The patient subsequently recovered well. Out of 5 patients with complications, there were 3 who underwent radical hysterectomy and all of the 5 had BPLND. There were no cases of mortality.

The hospital stay was calculated in all cases starting from the admission to initial discharge after surgery. The cases with complications and readmission were excluded. The results were that the uncomplicated robotic surgery hospital stay appeared to be shorter 1.3 days compared to the uncomplicated laparoscopic group, 2.5 days.

## 4. Discussion

The introduction of any new technology implies a learning curve experience which certainly applies to robotic surgery. The important issues in establishing a robotic surgical program are associated with organisational challenges, training, team building, and cost measured against benefit to the patient, surgeon, and institution.

Since the beginning of robotic surgery, various morbidity figures compared to both laparoscopic and open surgery have been reported [4–6, 9–12].

In 2011, Lim et al. compared the learning curves and associated morbidity in laparoscopic and robotic hysterectomy with lymphadenectomy. Less blood loss and a lower rate of conversion to the open procedure were reported with 0.8% intraoperative complication for robotic and 5.7% for laparoscopic procedures and postoperative complications were 4% for robotic and 12.3% for laparoscopic procedures. Readmission was reported as 8% for robotic and 3% for laparoscopic cases. The days of hospitalization of robotic and laparoscopic procedure were 1.5 ± 0.9 and 3.2 ± 2.3.

The mean operative time was 147.2 ± 48.2 and 186.8 ± 59.8 for robotic and laparoscopic procedures, respectively. A detailed comparative analysis of the learning curve showed that 24 cases are required to achieve proficiency with robotic procedure and 49 for laparoscopic one. The different robotic learning curve as suggested by the authors may be explained by the previous laparoscopic experience in MAS. The authors questioned the similarity of the learning curve for an “open surgeon” working towards the robotic proficiency [13].

Chandra et al. showed that novices demonstrated consistently better performance using robotics when compared to a standard laparoscopic setup [30]. Pilka et al. in their small series reported gradually shortening operation time, recovery time, and lowering blood loss within the “learning robotic curve” which was stated as less than 20 cases [31].

In 2012, Backes et al. retrospectively reported short and long term morbidity in 503 patients who underwent robotic assisted hysterectomy and lymphadenectomy (in 92.6%) [32]. In their study, median length of stay was one day. 6.4% of cases were converted to laparotomy. There were 1.6% of intraoperative complications, 7.6% patients developed major postoperative complications, and 3% required a transfusion in the 30-day perioperative period.

In 2013, Dubeshter et al. in their analysis showed that the hospital stay was 1.6 days for robotic and laparoscopic hysterectomies compared to 1.7 days for vaginal hysterectomy and 3.9 days for abdominal. The adjusted mortality rates for abdominal (0.20%), laparoscopic (0.03%), robotic (0.07%), and vaginal (0.04%) hysterectomies were not significantly different [3].

Lee et al. found that 78% of their series of robotic hysterectomies had successful same-day discharge [33].

Cardenas-Goicoechea et al. retrospectively compared 187 cases robot-assisted and 245 cases of laparoscopic staging for endometrial cancer. The overall rate of intraoperative complications was similar in both groups (1.6% versus 2.9%), but the rate of urinary tract injuries was statistically higher in the laparoscopic group (2.9% versus 0%). Patients in the robotic group had shorter hospital stay (1.96 days versus 2.45 days) but an average 57 minutes longer surgery than the laparoscopic group (218 versus 161 minutes). There was less conversion rate (0.5% versus 4.1%) and estimated blood loss in the robotic than in the laparoscopic group [34].

In 2014, Wechter et al. correlated postoperative complications with surgical variables in robot-assisted gynaecological surgery. The overall postoperative complications were reported as 18.4%, intraoperative, 3.2% (making a total rate of 21.6%), and conversion to laparotomy 3.2%. Complications that were Clavien-Dindo grade 3 (requiring surgical, endoscopic or radiological intervention [35]) or higher occurred in 5.2% [36].

However, the true incidence of complications associated with robotic surgery was questioned by Cooper et al. who in August 2013 published data from FDA (Food and Drug administration) device-related complication database, LexisNexis (the legal database), and PACER (Public Access to Court Electronic Records). They identified robotic surgery-related complications over a 12-year period. There were more than a million robotic cases performed over that period of time across all the surgical specialties. A total of 245 events were reported to the FDA during the study period, including 71 deaths and 174 nonfatal injuries. However, there were a number of cases identified from legal database and media reported adverse events which were not reported to FDA. These data made up a “sampling” of a large but unknown number of unreported or misreported adverse incidence associated with da Vinci surgery [37].

We focused our analysis on major perioperative complications. We found in our series that robotic cases on the “learning curve” had significantly higher incidence of complications with intraoperative 4.5%, postoperative 18.2%, and readmission 18.2%. The overall complications in the robotic “learning curve” group were 22.7% compared to 4.5% in established laparoscopic surgery group which was taken as a “baseline” complication rate for our institution.

It is noted that most of the complications were infective complications and all of them were in the cases of pelvic lymphadenectomy. It is worth noting that there were different energy sources available in our institution as in many others: for laparoscopic surgery, advanced haemostatic devices such as Harmonic Ace [38] and pulsed bipolar diathermy [39] and for robotic surgery, monopolar and standard bipolar diathermy. The discrepancy between the complication rate in our data between robotic and laparoscopic arm could be explained by the variable haemostatic abilities and tissue damage caused by the different energy devices. There is no evidence that the procedures done with different energy sources are comparable [40]. Also in further analysis associated with learning curve and structuring the training, it is important to bear in mind that pelvic lymphadenectomy has its own learning curve [13]. In addition, it is not unreasonable to suggest that extended antibiotic prophylaxis use might be considered in this situation.

Compartment syndrome is a rare complication of the surgery due to specific positioning of the patient [41–43]. The compartment syndrome in upper and lower extremities in the robotic surgery was previously reported [44, 45]. It may be explained not only by the fact that the robotic surgery requires unique positioning of the patient but also that the position cannot be easily changed after the da Vinci system is docked. Furthermore, the opportunity for reassessment of the situation may be limited due to relative remote position of the surgeon. In view of that further research should be done in identifying the measures to prevent this devastating complication.

Our study is small case series but aimed at the impact of learning curve of new technique on outcome in the situation when established approach already exists with reasonable safety profile. As the reported morbidity is potentially higher in the first cases compared to the established laparoscopic surgery, there is the ethical question of informing the patients that their case in on the “learning curve.”

Despite of small numbers, the time of surgery in our series was comparable with that reported previously [13, 34]. The surgical time for robotic hysterectomy and BSO without lymphadenectomy was higher comparatively to the laparoscopic cases, which is in agreement with previous data [46]. That may be due to the increased time of set up for robotic surgery, lack of haptic feedback, and the influence of the learning curve. Our feeling is that straightforward total laparoscopic hysterectomy in nonobese patient can be performed safely and in less operative time compared to robotic hysterectomy when performed by appropriately trained surgeons.

In our series, we also report shorter hospital stay in uncomplicated robotic cases. The difference could be explained by less pain experienced by the patients [47] possibly due to less abdominal distension used due to the possibility to perform the surgery with lower intra-abdominal pressure. Also, it could be possible that as robotic surgery was a new procedure to the hospital and there was a focus on the early discharge in the attempt to compensate for the expensive disposables and support the robotic programme development.

Another important point of discussion is the perception that robotic surgery is “one man” surgery. It is vital to emphasize that it is not. Substantial support from the robotic theatre team throughout the procedure is required to maintain safety. Zullo et al. in 2014 in their safety culture study in the robotic gynaecological operating room showed that the highest quality of communication and collaboration was reported by surgeons and surgical technicians with only adequate levels with other positions [48]. We have not conducted a formal assessment of the team work, but we observed that the team attention tends to decrease during the case/theatre list and the program implementation. That may be explained by the surgeon being positioned relatively remotely and not maintaining the usual direct contact with the rest of the team. Team training is important; however, our feeling is that to perform robotic surgery safely, there should be a “surgical assistant” that is able to control events inside theatre while the surgeon is immersed in the da Vinci console.

A randomised clinical trial is required to assess the benefit for the patient, society, and the surgeon in addition to creating a national centralised electronic database of MAS (including the robotic) which would allow collecting the information prospectively and identifying the trends.

## 5. Conclusion 

In our hands, robotic surgery is comparable to laparoscopic surgery in blood loss; however, the hospital stay in uncomplicated cases appears to be longer in the laparoscopic arm. Surgical robotic time is equivalent to laparoscopic in complex cases but may be longer in cases not requiring lymph node dissection. The robotic surgery team learning curve may be associated with a higher rate of morbidity in our hands; this was associated with the incorporation of lymphadenectomy in the hysterectomy procedure. A further research on the benefits to the surgeon is needed to clarify the whole picture of this versatile novel surgical approach.

## Figures and Tables

**Table 1:** A comparison of two consecutive series.

	Robotic (*n* = 22)	Laparoscopic (*n* = 22)
Age (mean)	55	69
BMI	29.3	28.06
TH + BSO mean surgical time (min)	151.2 (*n* = 13)	126.23 (*n* = 19)
TH + BSO + BPLND mean surgical time	178.3 (*n* = 4)	176.5 (*n* = 2)
RH + BPLND mean surgical time (min)	263.6 (*n* = 5)	264.0 (*n* = 1)
Conversion to laparotomy	None	None
Blood loss <100 mL	100%	100%
Complications total	22.7% (*n* = 5)	4.5% (*n* = 1)
Intraoperative complications	4.5% (*n* = 1)	0%
Postoperative complications	18.2% (*n* = 4)	4.5% (*n* = 1)
Readmission	18.2% (*n* = 4)	4.5% (*n* = 1)
Return to theatre	18.2% (*n* = 4)	4.5% (*n* = 1)
Blood transfusion	None	None
Hospital stay in uncomplicated cases (days)	1.3	2.5

**Table 2:** Complications in the robotic arm.

		Type of surgery	Time of presentation	Comorbidities	Management	Outcome
1	Infected pelvic haematoma	TH + BSO + BPLND	Day 5	Obese	Antibiotics	Recovered

2	Infected pelvic haematoma	TH + BSO + BPLND	Day 14	Morbidly obese	Antibiotics	Recovered

3	Infected pelvic haematoma	RH + BPLND	Day 3	Heavy smoker	Antibiotics/laparoscopic wash out by the emergency gynaecological team	Recovered

4	Anterior compartment syndrome of the lower leg	RH + BPLND	30 min	Sickle cell trait	Operated by plastic surgeons team	Recovered

5	Bilateral retroperitoneal abscess	RH + BPLND	Day 15	Immunocompromised patient	Antibiotics/bilateral radiological drainage/subsequent emergency surgery by colorectal team	Recovered
